# Obstetrical Follow-Up in Pregnancies After Radical Trachelectomy—Our Case Series and Proposed Cervical Length Measurement Protocol

**DOI:** 10.3390/jcm14145149

**Published:** 2025-07-20

**Authors:** Șerban Nastasia, Adina-Elena Nenciu, Adrian Valeriu Neacșu, Manuela-Cristina Russu, Nicoleta-Adelina Achim

**Affiliations:** 1 Department of Obstetrics and Gynaecology , “Carol Davila” University of Medicine and Pharmacy, 020021 Bucharest, Romania; serban.nastasia@umfcd.ro (Ș.N.); adina-elena.afloarea@drd.umfcd.ro (A.-E.N.); manuela.russu@umfcd.ro (M.-C.R.); nicoleta-adelina.achim@drd.umfcd.ro (N.-A.A.); 2 Department of Obstetrics and Gynaecology, “Dr I. Cantacuzino” Hospital, 020021 Bucharest, Romania

**Keywords:** pregnancy after radical trachelectomy, latent shortening, forced shortening, cervical cancer, fertility spearing treatment

## Abstract

**Background/Objectives**: Obstetrical monitoring following radical trachelectomy (RT) for cervical cancer is marked by the lack of a standardized protocol, which may lead to delays in the intervention for cervical shortening. In light of the typical cervical remodeling process that occurs at the onset of labor, we hypothesized that the onset of premature cervical shortening in patients who have undergone radiotherapy commences at the internal ostium. **Methods**: We introduced the concepts of internal distance (distance between internal cervical ostium and cerclage thread) and the latent shortening of internal distance, which is characterized as a painless reduction in the internal distance, serving as an early marker of preterm contractions, thus enabling timely tocolytic intervention. **Results**: Three patients spontaneously conceived after RT. They were obstetrically followed-up after RT, using a combined approach of transvaginal ultrasound cervical markers and cardiotocography. Active tocolysis was used if internal distance shortening was observed. All patients delivered term healthy babies. **Conclusions**: The consistent ultrasound evaluation of both internal and external distances permits the proactive diagnosis of premature contractions and enables swift therapeutic measures.

## 1. Introduction

Radical trachelectomy (RT) has emerged as an alternative to hysterectomy for early-stage cervical cancer patients (tumor ≤ 2 cm, FIGO stage < IB2, V0, pN0) who want to preserve their fertility. In the vaginal approach to RT, as developed by Daniel Dargent [1], nearly the entire cervix is removed, together with an upper vaginal cuff of about 2 cm, and the surrounding parametrial tissue and the uterine body is subsequently sutured to the upper part of the vaginal vault [1,2,3]. Pelvic lymphadenectomy is performed laparoscopically as part of the procedure [1,2,3].

Pregnancies after ART are at a very high risk of preterm birth. The main risks derive from the significant shortening of the cervix due to surgery, which decreases the tension-bearing capacity of the remaining uterus, especially in the second half of the pregnancy, when the lower segment starts to develop from the hypothetical isthmus located between the cervix and corpus uterus [4]. A short post-surgery cervix is also permissive with ascending germs from the vagina, leading to inflammation of the lower pole of the membranes and to the preterm premature rupture of membranes (pPROM) [5].

Another specific post-RT complication in the second half of pregnancy is genital bleeding from the residual venous plexus that surrounds the resection site and the lower pole of the uterus. This complication is especially reported by Japanese authors [4,6,7] and not in European populations [2].

While some studies propose management strategies for antenatal care following RT, a universally accepted protocol for obstetrical follow-up in such cases is still lacking [2,8,9].

There is a clinical need for sensitive markers to detect early cervical shortening in post-RT pregnancies, as traditional length-based cutoffs are often inapplicable due to the intrinsically short residual cervix. To address this gap, we introduce the concept of “internal distance”—the sonographic measurement from the internal cervical ostium to the cerclage thread—as a potential early warning indicator of cervical remodeling. When this distance shortens painlessly but progressively, we define it as “latent shortening”, hypothesizing it to be a precursor to preterm labor.

In this paper, we present a series of three pregnancies following RT, with a narrative focus on the development and application of this novel internal distance concept in prenatal surveillance, which developed as our awareness gradually increased. Our objective is to evaluate whether routine transvaginal sonographic monitoring of the internal distance, together with cardiotocography (CTG), might enable the earlier detection of subclinical contractions and support timely therapeutic interventions.

## 2. Materials and Methods

We report a three-case series of very early cervical cancer (FIGO stage ≤ IA2, V0, pN0) in young patients, para 0, with a strong wish to preserve fertility. In all patients, vaginal RT with laparoscopic pelvic lymph node excision was performed. We aimed to perform our procedures in a manner consistent with the oncological radicality of modified (type II) abdominal radical trachelectomy [9].

**Figure 1:**
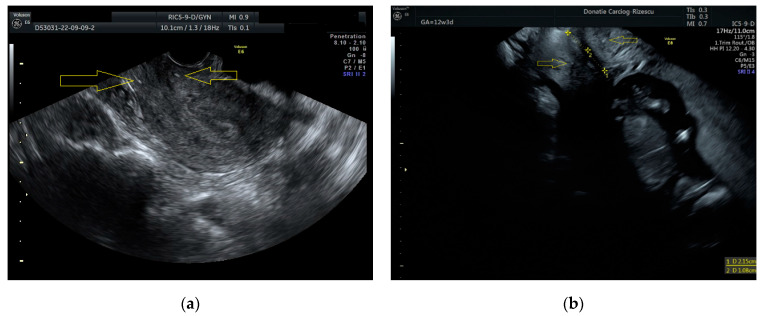
Patient no. 2. (**a**) Transvaginal examination of the non-pregnant uterus after RT and cerclage. The thread of cerclage is identified as two hyperechogenic spots (arrows). (**b**) Transvaginal ultrasound in the same patient (while 12 weeks pregnant) revealed a total cervical length of 21.5 mm, with an internal distance of 10.7 mm and external distance of 10.8 mm.

Obstetrical ultrasound follow-up in pregnant patients after RT was performed according to the usual screening protocol. Considering the normal process of normal cervical effacement, we considered that assessing the distance between the internal ostium and cerclage thread (called by us internal distance) would allow an earlier diagnosis of the onset of cervical shortening. In opposition with the internal distance, we defined the external distance as the distance between the cerclage thread and external ostium (Figure 1). By definition, post RT cervixes are short, so any tiny cervical shortening (of order of millimeters) could be indicative of the onset of premature labor.

In our series, we provided a narrative description of our increased awareness of the role of internal distance as a marker of early cervical shortening.

## 3. Results

In the interval of 2015–2024, four fertility-sparring surgeries for incipient invasive cervical cancer were performed in our clinical hospital. Four patients presented with high-risk cytology and positive colposcopy, with cervical carcinoma confirmed by cervical biopsy. Given the limited experience available at the moment of diagnosis, all patients were informed about the standard treatment and the fertility-sparring procedures (fertility-sparring treatment—FST) available in their cases. All patients chose vaginal RT with laparoscopic pelvic lymph node excision as FST, although cone biopsy and laparoscopic removal of the lymph nodes were also proposed. Prophylactic definitive cervical cerclage was performed at the time of intervention.

As a result of FST, pregnancy was spontaneously obtained in three patients (one pregnancy per patient); the fourth patient delayed the moment of conception. Pregnant patients were advised to limit physical activity and strictly observe sexual abstinence. Sexual intercourse was strictly forbidden, being considered a cause of uterine contractions, either through sperm prostaglandins or by promoting ascendent infections and lower-pole inflammation during pregnancy. The restriction of physical activity was indicated for all three patients, but bed rest was not recommended [10,11,12,13].

During pregnancy, the follow-up of the cervical cancer was performed according to existing protocols, using both Pap smears and a HPV test performed according to the time elapsed since RT [14,15].

A fetal ultrasound, performed according to the usual screening protocol, revealed no anomalies. All patients received prophylactically intravaginal progesterone and oral azithromycin. Tocolytics were prescribed in the case of uterine contractions.

Patient no. 1 had an uneventful pregnancy. She had only one hospital admission during pregnancy for bed rest. Obstetrical fetal follow-up was performed according to the usual obstetrical screening protocols, revealing no anomalies and a good fetal growth curve. Cervical length was assessed monthly via transvaginal ultrasound and remained unchanged throughout the pregnancy. The patient remained free of symptoms, and she had only one hospital admission during pregnancy for bed rest, as we were trying to somehow adhere to the available literature recommendations. Labor began spontaneously at 37 weeks of gestation, and the patient delivered by CS. During a retrospective analysis of the images, we were able to measure the internal distance and external distance, which remained unchanged throughout pregnancy. The measurements were not actively used for obstetrical follow-up in pregnancy.

For patient no. 2, obstetrical follow-up was performed according to the usual screening protocols, the same way as in patient no. 1. In the same manner, cervical length was assessed monthly with transvaginal ultrasound. At 17 weeks of gestation, we observed an asymptomatic reduction in the total cervical length. The cervix decreased from 21.5 mm at 12 weeks of gestation (Figure 1) to 18 mm at 17 weeks of gestation. The patient remained asymptomatic, and no uterine contractions were identified. However, an Arabin pessary was placed intravaginally, as we determined the total cervical length to be too short. During a retrospective analysis of the transvaginal ultrasound images, we were able to explain the total cervical length reduction by identifying an asymptomatic reduction in the internal distance from 10.7 mm at 12 weeks of gestation to 8 mm at 17 weeks of gestation. This internal distance was considered critically short and suggestive of the phenomenon we define as latent shortening of the inernal length. The patient had only one hospital admission during pregnancy for bed rest at the moment of Arabin pessary placement and remained free of contractions throughout the pregnancy. Cesarean section was performed at 38 weeks of gestation, before the onset of labor. This case raised our awareness regarding the utility of internal distance and external distance measurements; however, we did not make any decisions based on these measurements.

**Figure 2:**
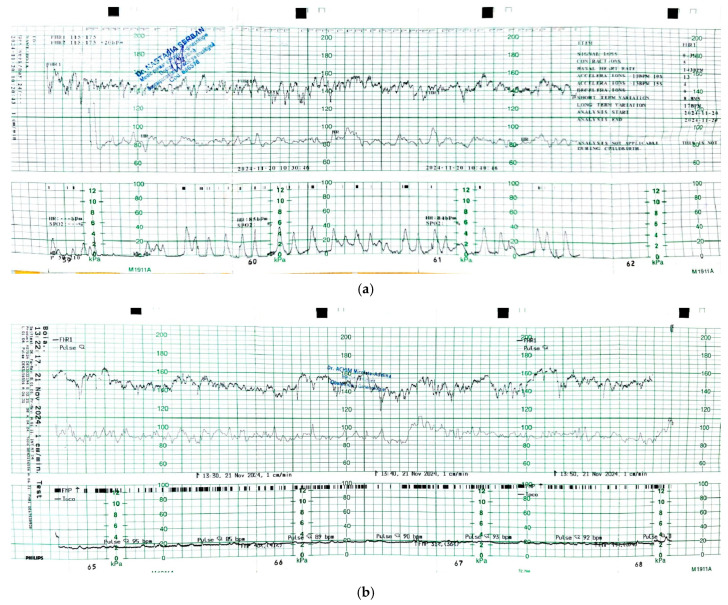
Patient no. 3. CTG recording at 24 weeks gestation allowed the early detection of premature uterine contractions (**a**). Cervical length was assessed through vaginal ultrasound and the distance cerclage thread—internal ostium decreased from 11 mm to 5 mm. The external distance remained unchanged (12 mm). Active tocolysis with nifedipine was started, stopping the contractions (**b**).

In patient no. 3, the concept of measuring the internal distance and external distance was actively used. The third patient was admitted monthly after week 20, due to contractility. Similar to the first two patients, cardiotocography (CTG) was performed starting at week 20, identifying uterine contractions (Figure 2a).

**Figure 3:**
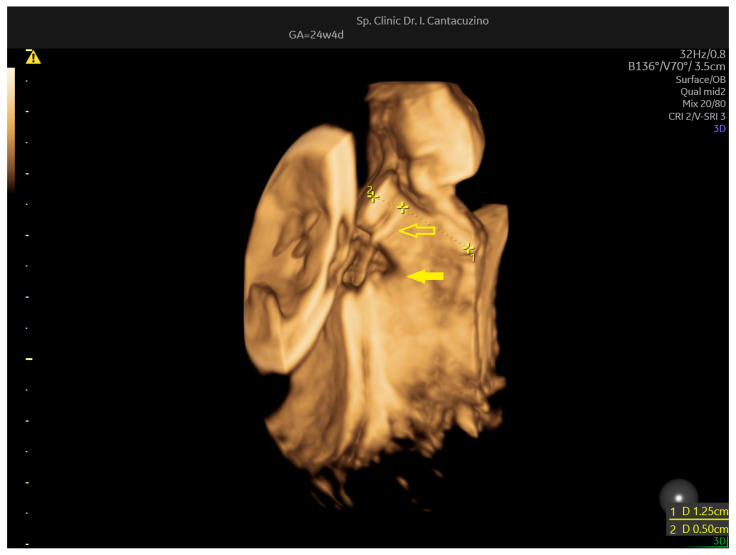
Patient no. 3. Transvaginal ultrasound at 24 weeks, triggered by asymptomatic contractility. Parasagittal section reconstruction revealing cerclage thread. Empty arrow points to cerclage thread. Internal distance was 5 mm. External distance was 12.5 mm. Solid arrow points to tendency for funneling below the thread.

The patient was asymptomatic; however, the uterine contractility triggered transvaginal ultrasound examination, which revealed a decrease in the total cervical length, from 21 mm to 17.5 mm, due to a decrease in the internal length (Figure 3). Active tocolysis with nifedipine was started, leading to the cessation of contractions; nifedipine was maintained for 2 weeks, along with bed rest in hospital. The monthly screening for urinary and vaginal infections in this patient revealed no vaginal or urinary infections.

**Figure 4:**
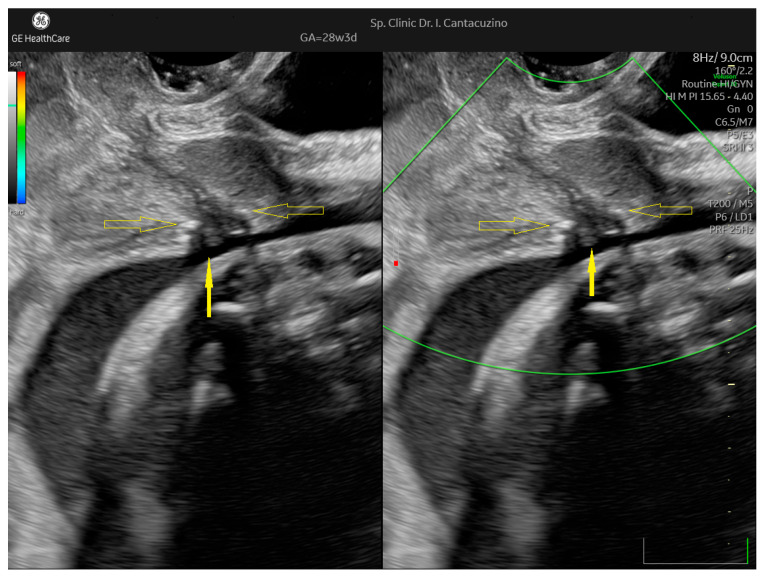
Patient no. 3. Transvaginal ultrasound at 28 W. Sagittal section. Empty arrows point to cerclage thread. Internal distance was 4.1 mm. External distance was 12 mm. Solid arrow points to tendency for funneling below the thread. Dilated vessels were not identified.

At 28 weeks of gestation, in an otherwise asymptomatic patient, transvaginal ultrasound identified a further reduction in the internal distance of about 1 mm, with a funneling shape in the internal ostium (Figure 4). Although a 1 mm distance could be interpreted as an intraobserver error, we interpreted this as a 20% reduction in the internal distance; thus, nifedipine was started again.

Of note, after 30 weeks of gestation, we were not able to further identify the cerclage thread by vaginal ultrasound. Cesarean section was performed at 38 weeks of gestation, before the onset of sustained contractions.

There were common recommendations for all pregnant patients. All pregnant patients were recommended to reduce physical activity and to strictly maintain sexual abstinence throughout the pregnancy. Sexual intercourse was strictly forbidden, being considered a cause of uterine contractions, either through sperm prostaglandins or by promoting ascendent infections and lower-pole inflammation during pregnancy. The restriction of physical activity was indicated for all three patients, but bed rest was not recommended.

During pregnancy, the follow-up for cervical cancer was performed according to existing protocols, using both Pap smears and a HPV test performed according to the time elapsed since RT. Fetal ultrasound, performed according to the usual screening protocol, revealed no anomalies.

For all three patients, medication included the daily intravaginal administration of 200 mg of prophylactic progesterone until week 35 [16]. Azithromycin was recommended in all three patients prophylactically, without any sign of cervical sludge or positive culture. Cardiotocography (CTG) was performed, starting at week 20, as a routine follow-up, regardless of whether the patients were asymptomatic or symptomatic. The rationale for CTG recording was to detect uterine contractions before they would have an effect on cervical length, but contractions were recorded only in the third patient (Table 1).

## 4. Discussion

While obstetrical ultrasound screening protocols for fetal anomalies are well defined, there is no current protocol regarding obstetrical surveillance of the remaining short cervix after RT during pregnancy [17,18,19,20,21]. As a result, the active treatment of cervical shortening could be delayed. Starting from the normal process of cervical shortening and effacement at the onset of labor, we postulated that the premature shortening of the cervix in RT patients will start at the internal cervical ostium. We observed that the early detection of cervical shortening is possible by assessing the distance between the internal ostium and cerclage thread (called by us internal distance). At the same time, we defined another marker, external distance, by measuring the distance between the cerclage thread and external ostium. By definition, both the internal and external distance are very short distances, so any tiny shortening could be of great significance (as in patient no. 3, where the 1 mm shortening of the cervix was interpreted as a 20% reduction in the internal distance, acknowledging interobserver variability as a limitation). Also, standardization and training for internal distance measurements are needed to minimize measurement errors. The shortening of the internal distance (called by us latent shortening) could be secondary to the presence of premature contractions, prompting tocolytic intervention, with cardiotocography as a part of this monitoring. Once the internal distance has vanished, the further shortening of cervix is possible only via the forced shortening of the external distance, with the tearing of the tissues anchoring the thread of cerclage.

The effect of the treatment of CIN and very early invasive cancer (stage IA1) was assessed in a systematic review performed by Kyrgiou et al. [22]. After analyzing 69 studies, including 65,098 pregnancies of treated and 6,292,725 pregnancies of untreated women, the authors concluded that the risk of preterm birth and extreme prematurity (less than 30 weeks) is significantly higher compared to women who had no treatment, and the risk increases with the depth of the cone excision, even for as little as 10–12 mm.

Radical trachelectomy is, by definition, an aggressive excisional method, leaving a small residual cervix. Attempts to establish a correlation between the length of the residual cervix after RT and the incidence of PPROM and premature delivery have been made. Using MRI-based measurements of the residual cervix in 29 patients with cervical cancer who become pregnant, Alvarez et al. [23] concluded that the incidence of PPROM and premature delivery are significantly increased when the residual cervix after RVT is less than 10 mm. Our proposed ultrasound marker, measurement of the internal distance, combined with CTG records, has the advantage of following the natural process of cervical shortening, while being readily available.

As pregnant patients after RT have very short cervixes from the beginning of the pregnancy, some authors recommend cervical length measurements every 2 weeks, starting from the second trimester of pregnancy (Knight LJ et al. [24], in a four-patient series), in order to early detect the risk of preterm delivery in otherwise asymptomatic patients. In our completely asymptomatic first two patients, ultrasound cervical assessment was performed monthly, whereas for the third patient, who had uterine contractility, vaginal ultrasound was performed every 2 weeks.

Current recommendations for management in pregnancy after RT are synthesized by Speiser D et al., including the absence of digital examination, vaginal pH measurements twice weekly, different techniques of cervical closure if the cervix is less than 1 cm, bed rest, the avoidance of physical strain and working, the absence of sexual intercourse [25], no elective dental procedures and no vaginal progesterone [2].

As our patients had remaining cervixes longer than 2 cm, we did not strictly adhere to this protocol, considering vaginal progesterone superior to oral progesterone. As a result, vaginal pH measurements were not performed. We adopted a different attitude, encouraging patients to perform vaginal douching when they felt the need, and performing vaginal cultures only once per trimester for two patients [17].

Our series has only a small number of very selected patients with a favorable prognosis, so we cannot make definitive recommendations. The absence of a control group makes it difficult to determine whether the proposed protocol truly improves outcomes, as standard obstetrical care for the control group has to be defined. Another limitation of our small study is the lack of a formal cost-effectiveness analysis: while our approach may raise costs, the early detection of preterm labor may reduce neonatal intensive care expenses.

While aware of our small number of cases, it is our opinion that internal distance could serve as an early marker for the cervical shortening process, which could allow every obstetrical case after RT to have an individualized follow-up.

## 5. Conclusions

Our experience highlights the importance of meticulous surveillance in pregnancies following RT. Importantly, we highlight the clinical utility of the “internal distance”—the sonographic measurement between the internal cervical ostium and the cerclage thread—as a potentially sensitive marker for early cervical shortening in post-RT pregnancies. This parameter may provide early warnings for the risk of preterm labor in a population already predisposed to cervical insufficiency due to surgical intervention. Monitoring the internal distance as a marker for latent cervical shortening enables early intervention, potentially improving pregnancy outcomes. Further studies are needed to establish standardized obstetrical management guidelines for RT patients.

## Figures and Tables

**Table 1:** Summary of data of case series.

	Patient No. 1	Patient No. 2	Patient No. 3	Patient No. 4
Age at pregnancy	32	37	NA (33 at operation)	28
HPV status	16	16	16	16
Cytology	HSIL	HSIL	HSIL	ASC-H
Pathology	Non-keratinizing squamous cell carcinoma, G2	Non-keratinizing squamous cell carcinoma, G2	Keratinizing squamous cell carcinoma, G1	Non-keratinizing squamous cell carcinoma, G2
FIGO stage	pT1a	pT1a2	pT1a2	pT1a1
Reinterventions		ERAD at 1 year after RT (HSIL, CIN-Tec Plus positive)		
Desire to immediately become pregnant	Yes	Yes	No	Yes
Onset of pregnancy (months after FST)	7	32		14
Cervical length (mm)	28	21.5		22.6
Cerclage thread—internal ostium distance (mm)	NA	10.7		10.1
Cerclage thread—external ostium distance (mm)	NA	10.8		12.5
Vaginal Arabin pessary	No	Yes		No
Amnionitis	No	No		No
Premature (<28 W) contractions	No	No		Yes
Steroids	No	No		Yes
Tocolitics	No	No		Nifedipine
Prophylactic intravaginal progesteron	Yes	Yes		Yes
Gestational age (weeks)	37	38		38
CS	Yes	Yes		Yes
Weight of the newborn (grams)	3000	2770		2920
Apgar score	9	9		8
Maternal complications	No	No		No
Fetal complications	No	No		No
Actual status	Free of disease	Free of disease	Free of disease	Free of disease

## Data Availability

The original contributions presented in this study are included in the article. Further inquiries can be directed to the corresponding author(s).

## Abbreviations

The following abbreviations are used in this manuscript:

RT	Radical trachelectomy
PROM	Premature rupture of membrane
ART	Abdominal radical trachelectomy
FST	fertility-sparring treatment
VRT	Vaginal radical trachelectomy
CS	Caesarean section
